# Modeling COVID-19 Outbreaks in Long-Term Care Facilities Using an Agent-Based Modeling and Simulation Approach

**DOI:** 10.3390/ijerph19052635

**Published:** 2022-02-24

**Authors:** Ali Asgary, Hudson Blue, Adriano O. Solis, Zachary McCarthy, Mahdi Najafabadi, Mohammad Ali Tofighi, Jianhong Wu

**Affiliations:** 1 Disaster and Emergency Management Area, School of Administrative Studies, York University, Toronto, ON M3J 1P3, Canada; 2 Decision Sciences Area, School of Administrative Studies, York University, Toronto, ON M3J 1P3, Canada; asolis@yorku.ca; 3 Laboratory for Industrial and Applied Mathematics (LIAM), Department of Mathematics and Statistics, York University, Toronto, ON M3J 1P3, Canada; zjm@yorku.ca (Z.M.); wuji@yorku.ca (J.W.); 4 Advanced Disaster, Emergency, and Rapid Response Simulation (ADERSIM), York University, Toronto, ON M3J 1P3, Canada; mirmahdi@yorku.ca (M.N.); tofighim@yorku.ca (M.A.T.)

**Keywords:** COVID-19, long-term care facilities, agent-based modeling, disease modeling, contact matrix

## Abstract

The elderly, especially those individuals with pre-existing health problems, have been disproportionally at a higher risk during the COVID-19 pandemic. Residents of long-term care facilities have been gravely affected by the pandemic and resident death numbers have been far above those of the general population. To better understand how infectious diseases such as COVID-19 can spread through long-term care facilities, we developed an agent-based simulation tool that uses a contact matrix adapted from previous infection control research in these types of facilities. This matrix accounts for the average distinct daily contacts between seven different agent types that represent the roles of individuals in long-term care facilities. The simulation results were compared to actual COVID-19 outbreaks in some of the long-term care facilities in Ontario, Canada. Our analysis shows that this simulation tool is capable of predicting the number of resident deaths after 50 days with a less than 0.1 variation in death rate. We modeled and predicted the effectiveness of infection control measures by utilizing this simulation tool. We found that to reduce the number of resident deaths, the effectiveness of personal protective equipment must be above 50%. We also found that daily random COVID-19 tests for as low as less than 10% of a long-term care facility’s population will reduce the number of resident deaths by over 75%. The results further show that combining several infection control measures will lead to more effective outcomes.

## 1. Introduction

The COVID-19 pandemic has passed through communities, affecting the most vulnerable people, especially the elderly [1]. Long-term care facilities (LTCFs) were hit hard by the pandemic, resulting in a significant number of deaths [2]. LTCFs have high contact environments with several high-risk individuals living very close to each other [3]. To reduce the spread of COVID-19 within an LTCF, several measures were introduced in addition to normal health and safety practices. These include stricter social distancing, scheduled and random testing of facility residents and staff, as well as the use of personal protective equipment (PPE) [2]. While these measures reduced the spread of COVID-19 infection, fatality rates in LTCFs remained at a higher level compared to the general fatality rate [2]. This calls for a better understanding of the measures listed above in terms of their capability to control the virus spread in an LTCF.

In the past couple of decades and prior to the onset of the COVID-19 pandemic, agent-based modeling (ABM) has been used in a good number of infectious disease epidemiological studies [4]. The traditional equation-based epidemiological models in earlier use proved to be problematic, as such models assume the population being modeled to be homogeneous. ABM allows the specification of characteristics and behaviors of individual agents, and the interaction of these agents with other agents and the environment based on a given set of rules [5]. Hernán [6] noted that ABM can be essential to an epidemiological study if empirical facts (data) based upon running an actual experiment are considered to be unattainable. However, El-Sayed et al. [7] compared the appropriateness of social network analysis and ABM as approaches for social epidemiology, noting that use of ABM “requires a balance between mechanistic rigor and model parsimony”.

Among others, ABM-based investigations have been conducted on outbreaks and transmission of influenza [8,9,10,11], H1N1 [12], SARS [13], tuberculosis [14], cholera [15], Ebola [16], measles [17,18], and dengue [19].

A growing number of COVID-19 related ABM studies have already been conducted [20,21,22,23]. Two earlier ABM-based investigations have been with respect to COVID-19 transmission in LTCFs [24,25]. Nguyen et al. [24] have used as base case a representative LTCF in Scotland, and they have examined interventions such as routine testing of staff, testing of new admissions, isolation of symptomatic residents, and restrictions of public visits. Smith et al. [25] evaluated the efficacy and resource efficiency of various surveillance strategies applied in a 170-bed LTCF in Northern France, including group testing, testing for symptoms and admissions, and random daily testing. Our work differs from previous studies [24,25] mainly because: (1) It develops an easy-to-use simulation tool that can be used for scenario analysis and planning; (2) it can be adapted and customized for LTCFs of different sizes; and (3) it can be further adjusted to be used for other types of communicable diseases.

This study has two main goals. First, it presents an agent-based simulation tool that models the spread of COVID-19 in an LTCF. The simulation tool presented in this paper informs decision-makers and gives insights about the best strategies to reduce the impact of LTCF outbreaks. Although agent-based modeling (ABM) has already been used to examine how COVID-19 affects a specific LTCF or a similar setting [24,25], the simulation model presented in this study allows for quick and easy adaptation to different facilities and infection control measures.

The second goal of this paper is to examine how infection control measures influence COVID-19 spread through an LTCF environment. More specifically, the effects of different levels of PPE effectiveness and testing frequencies were assessed in terms of their influence on the spread of the virus. In addition to these scenarios, a baseline scenario wherein a lack of response allows the virus to spread freely through the facility was also simulated. By comparing the number of residents who would decease under each scenario, the impact of different infection control measures on reducing the COVID-19 spread and fatality is understood.

## 2. Background

While most people who are infected by COVID-19 recover on their own, there are various factors that can influence how severely they are impacted by the virus [26]. For example, age, obesity, and diseases such as cancer and type two diabetes can increase the risk towards a more severe affection [26]. Among these factors, age is the most significant [1]. Throughout the pandemic, LTCFs have been a consistent hotspot for fatal COVID-19 outbreaks [27], with fatality rates significantly higher than the fatality rate in the general population [28]. Stall et al. [27] compared for-profit and not-for-profit LTCFs and found the fatality rate among infected residents in both types of facilities to be roughly 30%, although this rate can even be higher. Typically, the residents of these facilities are elderly who often experience several pre-existing health conditions [29]. For example, in Ontario, Canada, 82% of LTCF residents are 75 years old or above, and more than 50% are over 85 [30]. Thus, these residents are at higher risk of hospitalization and death from COVID-19 [29]. The physical layout and procedures also matter. The LTCFs typically have several residents living in close proximity inside a single building, giving a communicable disease such as COVID-19—which is transmitted by droplets—the opportunity to spread quickly among susceptible residents who are in close contact with an infected viral vector [27].

There are 627 LTCFs in Ontario. During the COVID-19 pandemic, LTCFs have been the epicenter of outbreaks in the province [2]. These facilities employ a total of over 100,000 staff, or 56,000 full-time equivalent (FTE) positions (as reported by LTCFs in 2018) that serve 78,000 residents with a variety of needs. This is a resident to FTE employee ratio of about 1.4:1 in which the number of staff, in terms of headcount, exceeds the number of residents. An average Ontario LTCF serves about 125 residents, but there is a significant variation in facility size [31]. About 40% of all Ontario LTCFs are small, with 96 beds or less, of which 58% are privately owned (for-profit), 24% non-profit/charitable, and 16% municipal [32].

The majority of LTCF staff in Ontario are personal support workers (PSWs) who account for about 58% of all care facility employees. The PSWs are responsible for assisting residents in their daily tasks. About 25% of LTCF staff are registered nurses, registered practical nurses, and nurse practitioners. Another 12% of them are allied healthcare providers (AHPs) such as physiotherapists, occupational therapists, and dieticians. Administrative staff account for about 1% of the overall staff of a facility. Cleaning and facility care staff numbers are less reported, but they seem to account for 1% to 4% of the facility staff members [32]. The staffing distribution is different due to the needs of the residents and thus, varies among facilities. On average, an LTCF resident receives around 3.73 direct hours of care per day, of which 2.3 h are from PSWs, which outnumbers nurses by one hour, and outnumbers AHPs by 0.4 h [31].

There is no uniform layout for LTCFs in the province of Ontario, though most facilities share a few common characteristics [33]. The number of residents per room varies from 1 to 4 in each home based on the home characteristics and the needs of the resident. It is not recommended, however, to have more than two residents in one room [33]. The vast majority of LTCFs have common areas such as dining and recreation spaces [34]. These spaces serve several residents concurrently and thus are a major cause of resident-to-resident contact. Visits take place in either the common sitting areas, recreation areas, or the resident’s room, depending on the facility setup and resident’s needs [33]. Ontario health guidelines require rooms and common spaces to be cleaned at least once per day [35]. To ensure the health and safety of residents, visitors generally need to check in and check out as they enter and leave [33].

## 3. Materials and Methods

### 3.1. Disease Simulation Model

An agent-based modeling tool, using the AnyLogic (version 8.7.3) modeling and simulation (M&S) platform, was developed for this study. To simulate an LTCF, seven agent types were defined: (1) resident, (2) personal support worker (PSW), (3) nurse, (4) allied health professional (AHP), (5) administrative staff (Admin), (6) housekeeper (HK), and (7) visitor (for an Overview, Design concept, and Description (ODD) format of the model see Table A1 in Appendix A).

**Figure 1:**
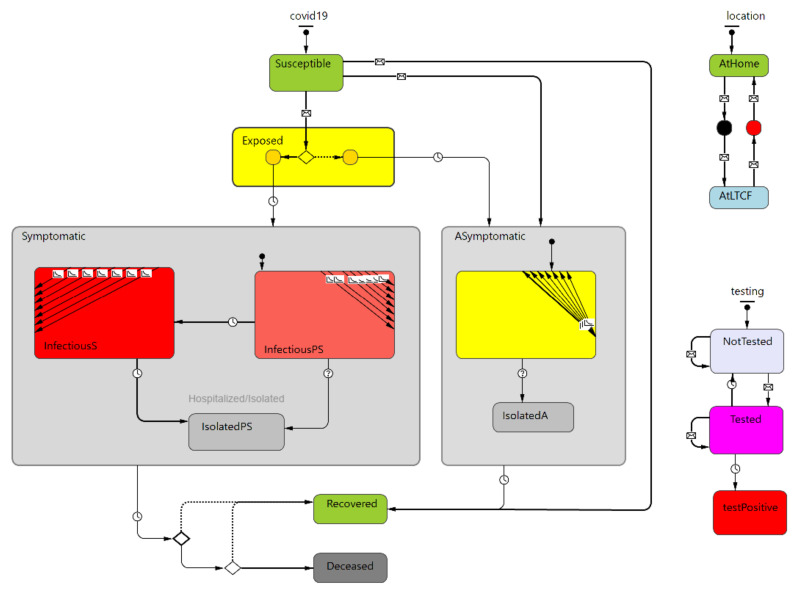
Outbreak simulation statecharts (disease transmission left, location top right, and testing bottom right).

To study the spread of an infectious disease, Rajagopal et al. [36] suggests a modified SEIRD model in which individuals are grouped as susceptible (S), exposed but asymptomatic (E), asymptomatic infected and symptomatic infected (I), recovered (R), and deceased (D), to represent the transmission of COVID-19. As in [21], our model groups the agents into susceptible, exposed, infectious (pre-symptomatic, symptomatic, and asymptomatic), recovered, and deceased. This modified version is able to better capture the COVID-19 as infected individuals can be with or without symptoms and symptomatic individuals can have different viral load and transmission rate before showing symptoms. The addition of pre-symptomatic individuals identifies the subgroup within the symptomatic group to differentiate between the transmission rates when the infectious person’s viral load is lower compared to the time when the individual is fully symptomatic with the maximum viral load. Agent statecharts are shown in Figure 1. (The simulation model does not currently take into account staff deaths. However, this may be added by modifying the corresponding statechart to allow staff in this group as well).

Agents’ testing conditions were defined by the testing statechart. If an agent was either randomly tested or had shown symptoms and was accordingly tested, that person’s state changed to the *tested* state. All agents who diagnosed with COVID-19 due to testing would be placed in isolation. If the test was negative, the individual stayed in the *tested* state during a certain period of test validity, and then its state changed back to *not tested* once the test results expired. PPE use was modeled by model parameters that moderate the PPE effectiveness in terms of reducing infection probability and the portion of individuals who wear PPE [37].

**Figure 2:**
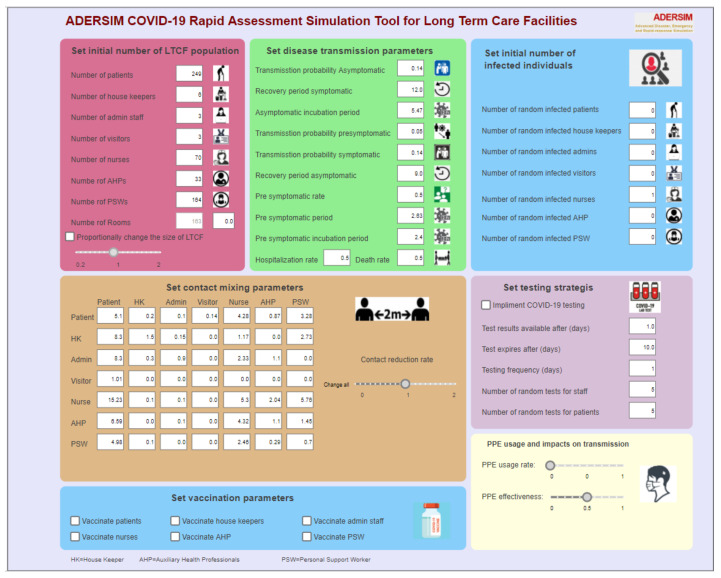
Parameter setting page for the outbreak modeling tool.

Before the actual simulation run, a parameter setup control panel (Figure 2) allowed the user to set the desired LTCF parameter values, disease transmission, the initial number of infected agents, contact matrix, PPE usage, testing, and vaccination. While vaccination is in the model boundary, we did not model it as an infection control measure. This is because we were modeling the initial stages of the pandemic (as of February of 2021) where there was not sufficient credible information on vaccination in LTCFs. Specifically, information on the adverse reaction rate, the average rate of exemption from vaccination for LTCF residents, and vaccine effectiveness among severely health compromised individuals were not available. Furthermore, the scenarios assessed in this paper were based on first wave data, while vaccines were not available. Thus, we were hesitant to include vaccination as it was not among the model data.

**Figure 3:**
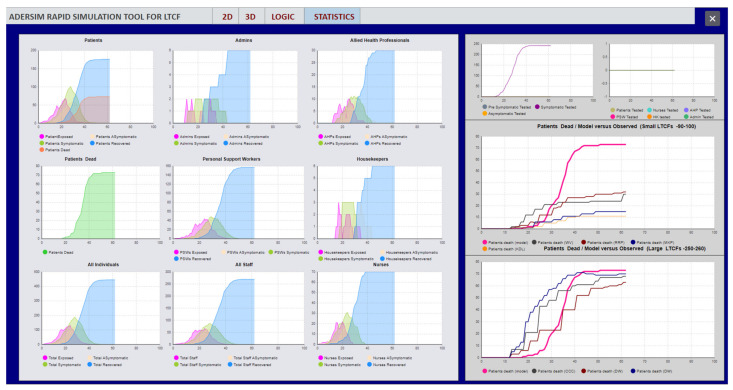
Example of output graphs from the model.

The simulation output is presented in a series of graphs in Figure 3. It displays the numbers of people who are recovered, symptomatic, asymptomatic, and exposed staff in the LTCF at any given time. There are additional graphs that show the number of residents who hospitalize and sometimes die due to the infection. While the model simulates several aspects of COVID-19 outbreaks in LTCFs, this paper focuses on resident deaths. This is because Ontario’s LTCFs COVID-19 infection data were not reported in a systematic manner and do not represent the actual state of the outbreak, particularly in the first wave of the pandemic from March to May 2020 [37]. As a result, verifying the accuracy of the model via initial infection data is not a viable option. However, reported deaths are more accurate and can be used to assess the model results.

### 3.2. Contact Matrix

We developed a contact matrix (Table 1) for LTCF based on the Duval et al. [3] study. This study used wearable sensors (Radio-Frequency Identification Devices [RFID]) to measure high resolution close contacts in a 200-bed LTCF, over a 4-month period. First, we utilized the established mixing matrix from the neurologic rehabilitation ward from [3] to partially inform the $C_{ij}$ entries. Next, we made use of the reciprocity condition $C_{ij}N_{i}=C_{ji}N_{j}$ to inform the entries $C_{ij}\;$ for $i=\mathrm{HK},\;j\in \;\left\{\mathrm{Resident},\mathrm{Nurse},\mathrm{AHP},\mathrm{PSW}\right\}\;$ as well as $C_{ij}$ for $i=\mathrm{Admin},\;j\;\left\{\mathrm{Resident},\mathrm{Nurse},\mathrm{AHP},\mathrm{PSW}\right\}$. These entries were calculated by using the formula derived from the reciprocity condition $C_{ij}=C_{ji}N_{j}/N_{i}$. Next, $C_{ij}$ for $i,j\in \left\{\mathrm{HK},\mathrm{Admin}\right\}$ and were informed by the transversal staff mixing matrix from [3]. The daily contact rates between different agent types were computed assuming that each resident has one visitor per week, and visitors do not interact with the LTC staff. The conservation law was used to identify the visitor contact rate with residents. Finally, a reciprocal correction was applied to ensure daily contacts are balanced.

Table 1 contains the calculated average number of distinct contacts, that one individual of a particular agent type (row) has with other individuals belonging to the same or another given agent group (column) over the course of a 24-h period. For example, a PSW agent has an average of 4.98 contacts with the resident agents, or resident agents has an average of 3.28 contacts with PSW agents.

## 4. Results

### 4.1. Baseline Model

Table 2 shows the parameter values used in the base simulation run. These parameters define how the disease spreads among individuals during the simulation. They are based on the parameters used in [21], which examined COVID-19 testing rates in schools using a similar agent-based modeling and simulation approach. In the baseline scenario, infection control measures were not implemented, and the disease was spread through a 249-resident LTCF unimpeded. The result of this baseline simulation provides a baseline against which the infection control measures’ effectiveness can be judged.

**Figure 4:**
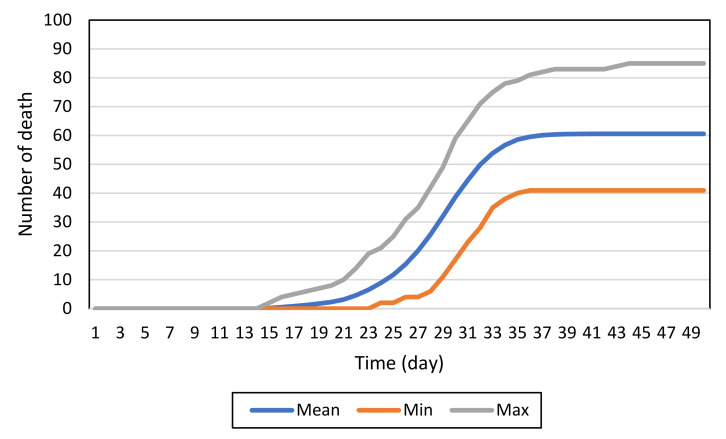
The death toll of 500 simulation runs of the baseline model.

Figure 4 shows the minimum, mean, and maximum simulated (predicted) death toll based on 500 Monte Carlo simulation runs of the baseline model. The horizontal axis represents days and the vertical axis represents the number of resident deaths. This base simulation used the parameters described in Table 2 with a 0.3 death rate. This simulation was set up for a facility with 249 residents, 164 PSWs, 70 nurses, 35 visitors, 33 AHPs, 6 housekeeping staff, and three administrative staff. COVID-19 is introduced into the facility by one infected nurse on day 1 of the simulation. The baseline model simulation predicts that, if COVID-19 spreads unimpeded under the specified parameters, there will be an average of 60.58 deaths after 50 days (minimum of 41 and maximum of 85 deaths).

If the model accurately predicts LTCF outbreaks, we can argue that the function and parameter settings in the model represent the actual conditions of LTCF outbreaks. Thus, conclusions drawn from that model can be applied to real-world situations.

To assess the model accuracy, we first compared the number of resident deaths at fifty days predicted by the model, against the number of resident deaths at fifty days observed in five LTCF outbreaks. The five facilities chosen were the ones that suffered from major outbreaks during the first infection wave in 2020. Facility sizes were close to the size of the LTCF based on which we created the contact matrix. To account for the varied sizes of the LTCFs, the parameters were set to reflect the actual number of residents as well as the average number of staff for an LTCF of that size in each particular LTCF. We assumed that PPE and daily COVID-19 test was limited in Ontario LTCFs at the time of the early outbreaks [38]. In each of the scenarios, the resident death rate was initially set at 0.3, as in the base model. This was used as the initial death rate because previous study suggests that the death rate in long-term care facilities with COVID-19 outbreaks is approximately 30% [39]. The results of these comparisons can be seen in Table 3 below. While it is difficult to say for certain the specific reason for the observed discrepancies there are several likely causes. The first is that in the real-world outbreaks the infection control measures in place may have changed over the course of the outbreak. This may have been due to supply issues or changes in the outbreak control strategy. This is difficult to capture as specific data is not available on changing infection control measures for each specific case. Another potential contributing factor is the possible that single events such as funerals, or additional COVID-19 introduction into the facility could have impacted the number of infected and subsequently the number of deaths.

### 4.2. Impacts of Infection Control Measures

To examine the effectiveness of PPE use and COVID-19 testing in reducing the spread of COVID-19 in LTCFs, we simulated three sets of scenarios: one per control measure, and one as a combination of both. Each scenario was simulated 500 times using a Monte Carlo model that randomly varied simulation parameters, as in [21]. In addition to the three infection control measure groups, the base model run was also presented as a baseline for comparison of control measure effectiveness. The first group of infection control scenarios examined how differing effectiveness of PPE use can influence the spread of COVID-19. The second group of infection control scenarios involved the administration of differing numbers of COVID-19 tests within the LTCF: 10, 20, and 40 tests per day, evenly split between the staff and the residents. The final group of scenarios combined both PPE use and COVID-19 testing to examine how much these measures would be effective when used together. These three scenarios consider (i) 50% effective PPE use and 10 COVID-19 tests per day, (ii) 75% effective PPE use and 20 COVID-19 tests per day, and (iii) 90% effective PPE use and 40 COVID-19 tests per day.

#### 4.2.1. PPE Effectiveness

**Figure 5:**
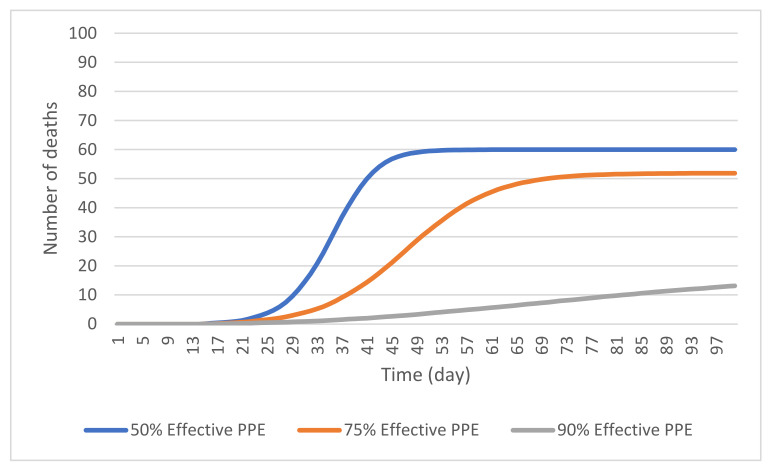
The cumulative number of resident deaths in a simulated outbreak with universal use of 50%, 75%, and 90% effective PPE.

To examine the effect of PPE on controlling the spread of COVID-19, three levels of PPE effectiveness were considered. Specifically, three scenarios regarding the consistent use of PPE at 50%, 75%, and 90% effectiveness were simulated. These scenarios were simulated with the assumption that PPE could be universally used within the facility. However, access to PPE in LTCFs was not the same during the early days of the pandemic. The cumulative number of resident deaths over the course of the simulation can be seen in Figure 5 (light blue-colored curve). To clarify the percent effectiveness of PPE represents the proportionate decrease in the likelihood of an individual to individual spread within the population. The use of 50% effective PPE reduced the rate of spread, evident in the 68.2% reduction in reported deaths at 25 days compared to the base model. It did not, however, reduce the cumulative number of deaths across 500 simulation runs, of 59 deaths in this scenario and a mean of 61 deaths in the base model. This suggests that a 50% level of PPE effectiveness would not significantly reduce the number of deaths.

Having 75% effective PPE reduced both the rate of spread of the disease and the total number of deaths at 50 days. Figure 5 shows the cumulative number of resident deaths when 75% effective PPE is used, averaged across 500 simulation runs. The cumulative number of resident deaths reported in this scenario at 50 days was 31, which represents a reduction of 49.1% compared with the baseline scenario. At 100 days the 75% effectiveness scenario showed a total of 52 resident deaths. This is only a 13.5% reduction from the previous scenario suggesting that this level of PPE effectiveness is less effective long term.

The 90% PPE effectiveness scenario showed a significant reduction in the cumulative number of resident deaths at 50 days compared to the previous PPE effectiveness scenarios at 50% and 70%. Figure 5 (gray-colored curve) shows the daily mean number of resident deaths with 90% effective PPE being used. The cumulative number of deaths, averaged over 500 simulation runs, at 50 days was only about 3, indicating a 95.1% decrease in resident deaths compared to the base model. This also represents a reduction of 90.3% from the mean number of deaths in the 75% PPE effectiveness case at 50 days. At 100 days, the 90% effectiveness scenario showed 13 resident deaths. This is still a large reduction from the previous conditions and base model. These results are consistent with previous literature which suggests that there is a non-linear relationship between the mask use efficiency and the infectiousness of the COVID-19 virus [40] that suggest that the current model produces results that match the real-world observations.

#### 4.2.2. Random Testing

**Figure 6:**
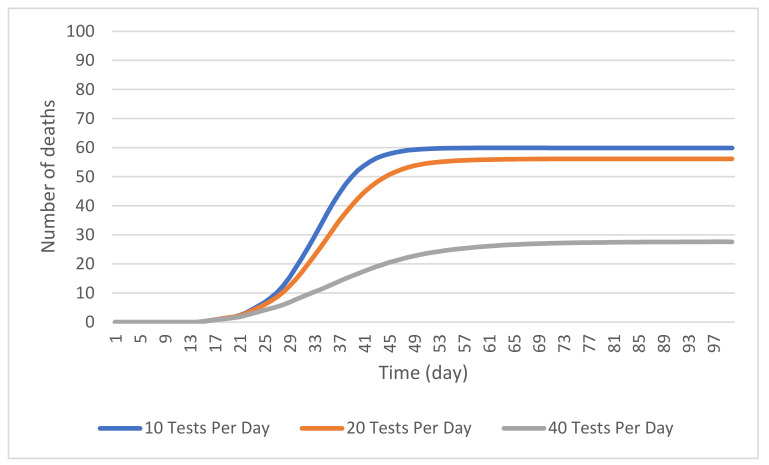
The cumulative number of resident deaths in a simulated outbreak with 10, 20, 40 random tests of staff and residents.

Three scenarios were simulated to examine the effect of testing and self-isolation on the number of resident deaths in 50 days. Figure 6 shows the outcome of these scenarios. Where a total of 10 COVID-19 tests are administered to a combination of staff and residents per day, a cumulative number of 59 residents die after 50 days, as averaged across 500 simulation runs. This does not differ much from the cumulative number of 61 at 50 days in the baseline scenario. At 25 days, however, this scenario showed a mean of 6 resident deaths that indicates a 50% decrease from the base model mean of 12 resident deaths at 25 days, suggesting that introducing this number of tests slows the rate of infection. However, the very small reduction in the cumulative number of resident deaths suggests that this testing level does not significantly reduce the number of deaths expected in an outbreak. This trend was consistent at 100 days.

With 20 tests per day, the cumulative number of predicted deaths at 50 days was reduced to 54. Introducing testing at this level did decrease the average number of resident deaths by 11.5%. However, the wide distribution of results across the 500 simulation runs makes it difficult to conclude if this testing level is actually effective at reducing the severity of an outbreak. That being said, the 100-day cumulative number of about 56 resident deaths suggests minimal effectiveness.

Increasing the number of tests to 40 per day produced a noticeable decrease in the number of resident deaths at 50 days. The results, averaged across 500 simulation runs, showed a cumulative number of 23 resident deaths, representing a 62.3% reduction from the corresponding number in the baseline model. This large decrease suggests that random testing at this level would reduce the number of deaths in a COVID-19 outbreak. Given that this scenario involves only testing 7.6% of the total 525-person LTCF population (residents and staff) on a daily basis, it makes a compelling case for the use of random testing as an effective infection control tool. At 100 days, this testing scenario produced a mean of about 28 resident deaths. This is a 50% reduction from the previous scenario.

#### 4.2.3. Combined Control Measures

**Figure 7:**
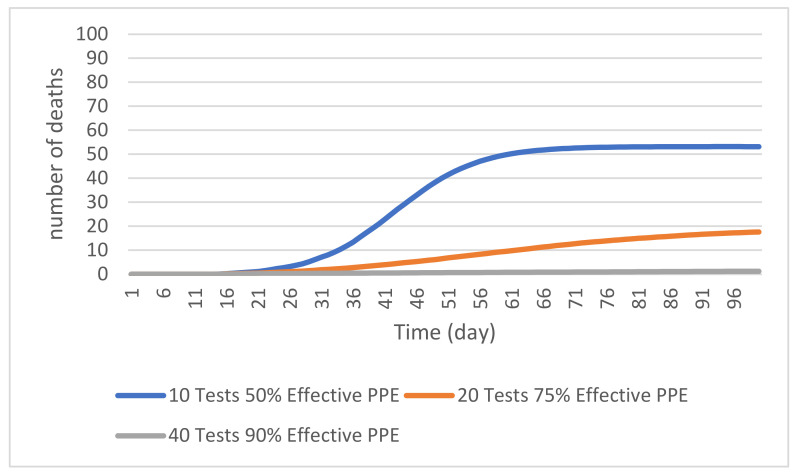
Cumulative number of resident deaths with different PPE effectiveness rate and daily number of random testing of residents and staff.

Figure 7 shows the number of resident deaths reported under the scenarios involving three different combinations of PPE effectiveness and random COVID-19 testing. Results show that, with 50% PPE effectiveness and 10 COVID-19 tests per day, the cumulative number of resident deaths at 50 days reach an average of 40 across 500 simulation runs. This is a 34.4% reduction from the mean cumulative number of 61 resident deaths in the base model, as well as a reduction of 32.2% from the 50% PPE effectiveness scenario and a 32.2% reduction from the 10 test per day scenario. The decrease in the cumulative resident deaths supports a conclusion that the infection control measures, when used together, will be more effective in reducing the resident deaths caused by COVID-19. This suggests that when control measures are taken individually at lower levels, they do not have much effect on the simulated cumulative number of resident deaths compared to the base model. In practical terms, the result of this combined measures scenario suggests that several minimally effective infection control strategies, when used together, can be more effective than when used individually.

The next scenario involves the use of 75% effective PPE and 20 tests per day. The simulation over 500 runs predicted that there would be a mean cumulative number of about 6 resident deaths at 50 days. This represents an 85% reduction from the mean cumulative number of resident deaths at 50 days under the first mixed measures scenario and a 90.2% reduction from the base scenario at 50 days. The current scenario also leads to an 80.6% reduction in the mean number of deaths at 50 days under the 75% PPE effectiveness scenario and an 88.9% reduction from the 20 tests per day scenario at the 50-day mark. This again suggests that it is better to combine infection control measures even if these measures are not fully effective individually. Even considering that the number of deaths at 100 days in this scenario was about 18, this is still much lower than the control and first scenario. Finally, with a combined use of 90% effective PPE and 40 tests per day, the simulation shows the greatest reduction in the cumulative number of resident deaths, with a mean of less than 1 resident death at 50 days. This scenario reached a maximum of about 1 resident death at 100 days suggesting that there is almost no transmission.

## 5. Discussion

This study had two main goals. First, to develop and present a simulation tool that models/characterizes the spread of COVID-19 in an LTCF. With respect to this goal, we developed, using the AnyLogic version 8.7.3 simulation software, an agent-based modeling tool based on a modified SEIRD model as earlier used in [21] in simulating preventative COVID-19 testing in schools. A baseline model which did not involve the application of infection measures was first tested on a small sample of five LTCFs that experienced COVID-19 outbreaks to set the base run corresponding with the early days of the pandemic. PPE and COVID-19 test kits were not yet readily available at that time. We performed, using the baseline model, simulations of 500 runs to predict the number of resident deaths, and compared the simulated death tolls with the observed numbers at the LTCFs in our sample. We found that adjusted base parameters would predict death tolls accurate enough to fall within a 0.1 variation of the observed death toll. The accuracy of the model outputs can be further improved with the use of parameter settings specific to each of the individual LTCFs that are currently known to the modeling team. This model is a predictive tool for how the disease would affect an LTCF under different epidemiological transmission/infection and mortality rates and different implemented control measures.

The second goal of the study was to examine how infection control measures can potentially impact the spread of COVID-19 in an LTCF. Not surprisingly, higher levels of PPE effectiveness or COVID-19 random tests were found to be more effective in reducing the simulated death tolls. In both the PPE effectiveness and test rate scenarios, the lowest level simulated in each case led to a minimal effect on the number of resident deaths in comparison with the observed number. However, when they were implemented together, they significantly reduced the death toll. In practical terms, this is an interesting result because it suggests that minimally effective control measures may become effective when implemented together. These scenarios also suggest that daily testing and isolation of less than 10% of the total population of the LTCF can reasonably decrease the number of deaths associated with the disease. As rapid testing becomes more available and accurate, daily COVID-19 test screening becomes a more viable option. Vaccination will also play a crucial role in reducing the number of COVID-19 deaths in an LTCF [41]. Non-pharmaceutical measures, however, such as PPE and testing will likely remain relevant throughout the vaccination process and afterwards, depending on how effective the vaccine turns out to be [41]. These results also support the idea that the infection control effects of wearing PPE are accelerated as PPE efficiency increases non-linearly [40]. This is helpful when PPE effectiveness in a facility is high because there would be a decline in the number of associated COVID-19 deaths. However, when PPE effectiveness is low, the benefits of PPE use become negligible.

Based on the results of this study, PPE effectiveness must be above 50% for a meaningful reduction in COVID-19 deaths. The best-case scenario presented as the third scenario in which both control measures were combined, suggests that it is theoretically possible to nearly eliminate COVID-19 deaths of residents in an LTCF by high levels of multiple infection control measures such as PPE use and random COVID-19 testing combined. Further research is needed to understand how social distancing and other control measures will influence the number of deaths in an LTCF.

The ability to understand these outcomes informs decision-makers regarding issues such as PPE allocation, social distancing measures, and testing programs. This model allows the user to adjust specific disease parameters. This means that model users can simulate the virus transmission and effects of epidemiological diseases other than just COVID-19. We learned from this most recent pandemic that having tools such as this model available helps to better prepare for, and mitigate the effects of, future global health risks faced by communities such as LTCF.

This modeling tool also has some academic applications. Similar models have been used in this capacity in the past [21]. The use of the current model provides a low-cost, readily available means of examining the vulnerability of LTCF. Future research in this area should focus on adapting the model to a broader range of facility sizes that are very different from an average LTCF. This would both improve the practical usefulness of the model as well as provide an opportunity to test the model robustness when considering smaller populations and different physical layouts. Further validation of the model would also be valuable as more data becomes available from other LTCF outbreaks under different conditions. Specifically, better data regarding the timeline of implementation of disease control measures would allow for better validation of the outcomes predicted by the model associated with these measures. Another area where this work could be expanded is to focus on vaccination and the role that this would play in controlling a disease outbreak. The impact of rapid testing as a screening option could also be examined. Fortunately, all of these potential future research topics are within the capability of the model, which could provide a platform for further inquiry and improvement.

## 6. Conclusions

Our study showed that agent-based modeling can be applied to develop a simulation tool for communicable disease management in LTCF. We developed and tested a simulation tool based on the attributes of COVID-19 in the province of Ontario (Canada) and disease outbreaks in LTCF. Using a detailed contact mixing matrix, the simulation tool is able to estimate potential impacts of disease outbreaks on LTCF residents and different staff. In particular, this tool can be used to assess the impacts of different public health measures such as testing and personal protection measures. While the simulation tool developed in this study works best for LTCF of specific size, where a contact matrix was available, it is expected that advancement in contact tracing and contact matrix generation technologies such as wearable devices, mobile applications, and artificial intelligence will soon enable access to contact matrices for LTCF of different sizes.

## Figures and Tables

**Table 1:** Contact matrix for the simulated long-term care facility.

	Resident	HK	Admin	Visitor	Nurse	AHP	PSW
**Resident**	5.1	0.2	0.1	0.14	4.28	0.87	3.28
**HK**	8.3	1.5	0.15	0	1.17	0	2.73
**Admin**	8.3	0.3	0.9	0	2.33	1.1	0
**Visitor**	1.01	0	0	0	0	0	0
**Nurse**	15.23	0.1	0.1	0	5.3	2.04	5.76
**AHP**	6.59	0	0.1	0	4.32	1.1	1.45
**PSW**	4.98	0.1	0	0	2.46	0.29	0.7

**Table 2:** Simulated COVID-19 parameters.

Parameter Name	Value (Unit)
Transmission Probability	14% (per each contact)
Symptomatic Recovery Period	12 (days)
Asymptomatic Incubation Period	5.47 (days)
Transmission Probability Pre-symptomatic	3% (per contact)
Transmission Probability Asymptomatic	14% (per contact)
Recovery Period Asymptomatic	9 (days)
Residents Death Rate	30%
Pre-symptomatic Period	2.63 (days)
Pre-Symptomatic Rate	50% (per person infected)
Pre-Symptomatic Incubation Period	2.4 (days)
Outbreak Progression Day (Day the Simulation Begins)	0

**Table 3:** Deviations between simulated and observed LTCF resident deaths (baseline model).

LTCF	Camilla		Forest		Downsview	Orchard		Seven Oaks	
Death Rate	0.3	0.4	0.3	0.2	0.3	0.3	0.4	0.3	0.2
Simulated Deaths	61	82	61	41	61	61	81	69	24
Observed Deaths	68	68	51	51	63	70	70	41	41
% Error	11.5	−17.1	−16.4	24.4	3.3	14.8	−13.6	−40.6	70.8

## Data Availability

Not applicable.

## Appendix A

**Table A1:** Overview, design concepts and details of ABMs.

Purpose	To develop a simulation tool for disease spread in long-term care facilities and examine the impact of different infection scenarios, public health measures including PPE usage, and COVID-19 testing
Entities, state variables, and scales	ABM consists of seven entities: (1) resident, (2) personal support worker (PSW), (3) nurse, (4) allied health professional (AHP), (5) administrative staff (admin), (6) housekeeper (HK), and (7) visitor, and each entity has several state variables Susceptible Exposed Symptomatic ◦ Infectious pre-symptomatic ◦ Infectious symptomatic ◦ Self isolated Asymptomatic ◦ Self isolated Recovered Diseased COVID-19: Not test COVID-19: Tested COVID-19: Tested quarantined At home At work
Process overview and scheduling	(1) Movement Human: Non-patient agents move between their home and workplace (long-term care facility) (2) A SEIR model is used for disease transmission All individuals are in a susceptible state at the start of the model Infection is triggered by one or more random infectious person(s) entering the system (3) Except patients, staff are going to long-term care facility during their shifts in a daily basis (4) COVID-19 testing state chart processes the random testing of agents
Design concepts Basic principles	The ABMs purpose is to model disease transmision in long-term care facilities based on residents distribution in the facility, close contacts between the residents and various staff working in the facility, and use of different public health meaures to control the disease
Interaction Details	There are interactions between patients with other human agents and among other human agents. The interactions are reflected in the contact matrix, see Table 1
Initialization	The simulation models long-term care facilities with a selected number of LTCFs residents and staff
Input data	(1) Contact matrix (Table 1) (2) Initial size of the LTCFs (3) Initial number of infected individuals
Parameters	The parameters of COVID-19 disease transmission as provided in Table 2
